# Effect of a recent parenteral dexamethasone and ketoprofen administration on the immunological diagnosis of tuberculosis in goats

**DOI:** 10.3389/fvets.2022.1042428

**Published:** 2022-11-10

**Authors:** Javier Ortega, Lucia de Juan, Iker A. Sevilla, Joseba M. Garrido, Álvaro Roy, Carlos Velasco, Beatriz Romero, Mercedes Domínguez, Bernat Pérez de Val, Carolina Nebot, José Luis Sáez-Llorente, Julio Álvarez, Javier Bezos

**Affiliations:** ^1^Departamento de Sanidad Animal, Facultad de Veterinaria, Universidad Complutense de Madrid, Madrid, Spain; ^2^VISAVET Health Surveillance Centre, Complutense University of Madrid, Madrid, Spain; ^3^Animal Health Department, NEIKER-Basque Institute for Agricultural Research and Development, Basque Research and Technology Alliance (BRTA), Derio, Spain; ^4^Unidad de Inmunología Microbiana, Centro Nacional de Microbiología, Instituto de Investigación Carlos III, Madrid, Spain; ^5^IRTA, Centre de Recerca en Sanitat Animal (CReSA, IRTA-UAB), Universitat Autònoma de Barcelona, Barcelona, Spain; ^6^Laboratorio de Higiene, Inspección y Control de Alimentos (LHICA), Facultad de Veterinaria, de Santiago de Compostela (Campus de Lugo), Lugo, Spain; ^7^Ministerio de Agricultura, Pesca y Alimentación, Madrid, Spain

**Keywords:** caprine tuberculosis, dexamethasone, ketoprofen, diagnosis, intradermal tuberculin test

## Abstract

Caprine tuberculosis (TB) is a zoonosis caused by members of the *Mycobacterium tuberculosis* complex (MTBC). Caprine TB eradication programmes are based mainly on intradermal tuberculin tests and slaughterhouse surveillance. Different factors may affect the performance of the TB diagnostic tests used in caprine herds and, therefore, their ability to detect infected animals. The present study evaluates the effect of the fraudulent administration of two anti-inflammatory substances, dexamethasone and ketoprofen, on the performance of the TB diagnostic techniques used in goats, as well as the suitability of high performance liquid chromatography (HPLC) for their detection in hair samples. The animals (*n* = 90) were distributed in three groups: (1) a group treated with dexamethasone (*n* = 30); a second group treated with ketoprofen (*n* = 30); and a third non-treated control group (*n* = 30). Both dexamethasone and ketoprofen groups were subjected to intramuscular inoculation with the substances 48 h after the administration of bovine and avian purified protein derivatives (PPDs), that is, 24 h before the tests were interpreted. All the animals were subjected to the single and comparative intradermal tuberculin (SIT and CIT, respectively) tests, interferon-gamma release assay (IGRA) and P22 ELISA. The number of SIT test reactors was significantly lower in the dexamethasone (*p* = 0.001) and ketoprofen (*p* < 0.001) groups 72 h after the bovine PPD inoculation compared with the control group. A significantly higher number of positive reactors to IGRA was detected within the dexamethasone group (*p* = 0.016) 72 h after PPD administration compared to the control group. Dexamethasone and ketoprofen detection in either hair or serum samples was challenging when using HPLC since these substances were not detected in animals whose skin fold thickness (SFT) was reduced, what could be an issue if they are used for fraudulent purposes. In conclusion, the parenteral administration of dexamethasone or ketoprofen 48 h after the PPDs administration can significantly reduce the increase in SFT (mm) and subsequently the number of positive reactors to SIT test.

## Introduction

Caprine tuberculosis (TB) is a chronic zoonotic disease caused by members of the *Mycobacterium tuberculosis* complex (MTBC), and principally *Mycobacterium tuberculosis* variant *bovis* (*M. bovis*) and *M. tuberculosis* variant *caprae* (*M. caprae*), being *M. caprae* the main causative agent in Spain (1). Tuberculosis is known to be a multi-host disease that affects a wide range of domestic and wildlife species and humans (2–5). Moreover, the presence of TB in livestock leads to economic losses owing to the reduction in milk yield and fertility, condemnations at slaughterhouses, restrictions on markets and the cost of diagnosis (6–8). In the case of Spain, whose caprine population is one of the largest in the European Union (EU) (9), certain regions have implemented regional eradication programmes (10). These caprine TB eradication programmes are based mainly on test and cull strategies using the single and comparative intradermal tuberculin (SIT and CIT, respectively) tests as the cornerstone of the ante-mortem diagnosis, and slaughterhouse surveillance (6). The interferon-gamma release assay (IGRA) has, in specific cases, also been used to diagnose TB in goats (6, 10, 11). Moreover, the prior diagnosis of TB in ruminants is considered a requirement for movements between Member States by the current European legislation (Regulation EU 2016/429). This legislation also considers the IGRA as an official test to allow movements of animals within the EU.

Both of these techniques, which are based on the cellular immune response, are valuable diagnostic tools, although they are not perfect in terms of sensitivity and specificity, and several factors could affect their performance (11–15). One of these factors is the administration of anti-inflammatory substances, which are, according to previous studies in goats (16) and cattle (17, 18), able to modify the results of official TB diagnostic tests carried out in ruminants. A recent study in goats demonstrated that a recent topical application of a corticosteroid (betamethasone) at the PPD injection site can significantly reduce the number of reactors to SIT/CIT tests (16). This effect was not observed on the IGRA and P22 ELISA results, and this finding was associated with the topical application of betamethasone (16). Nevertheless, previous studies have shown that the parenteral administration of dexamethasone reduces IFN-γ production in TB-infected cattle, resulting in false negative reactors to the IGRA (17, 18). These results therefore suggest that the administration of corticosteroids can interfere with the diagnosis of TB in ruminants in different ways depending on the administration route (16–18). The parenteral administration of anti-inflammatory substances could potentially be used with fraudulent purposes in order to interfere with the diagnosis of TB by inhibiting the local (SIT/CIT tests) and systemic (IGRA) inflammatory reactions in goats. However, to the best of the authors' knowledge, no previous studies have evaluated the effect of the parenteral administration of anti-inflammatory substances on the outcome of the TB diagnostic tests in goats. In fact, not only corticosteroids, but also other anti-inflammatory substances could be used for this purpose according to personal communication of official veterinary services of Spain. In this context, in the recent years the authorities of different regions in Spain have started to research the fraudulent use of different substances parenterally administered in order to modify the result of official TB diagnostic tests in ruminants (19). Moreover, it is important to highlight the difficulties involved in demonstrating this type of fraudulent activities due to the high variety of substances and administration protocols which can be applied in ruminants to modify the TB diagnostic tests outcome. The development and evaluation of methodologies, in order to establish effective protocols for the detection of these substances, are consequently of paramount importance. Therefore, the aim of the present study was to determine, for the first time, whether the parenteral administration of dexamethasone or ketoprofen 48 h after the PPDs administration interferes with the diagnosis of caprine TB by intradermal tests, and whether this protocol also affects the results of other diagnostic techniques such as IGRA and Ab-based tests. A specific protocol for the detection of dexamethasone and ketoprofen in serum and hair samples was also developed and applied in order to demonstrate the presence of these substances in the goats from the different experimental groups.

## Materials and methods

### Experimental design

**Figure 1:**
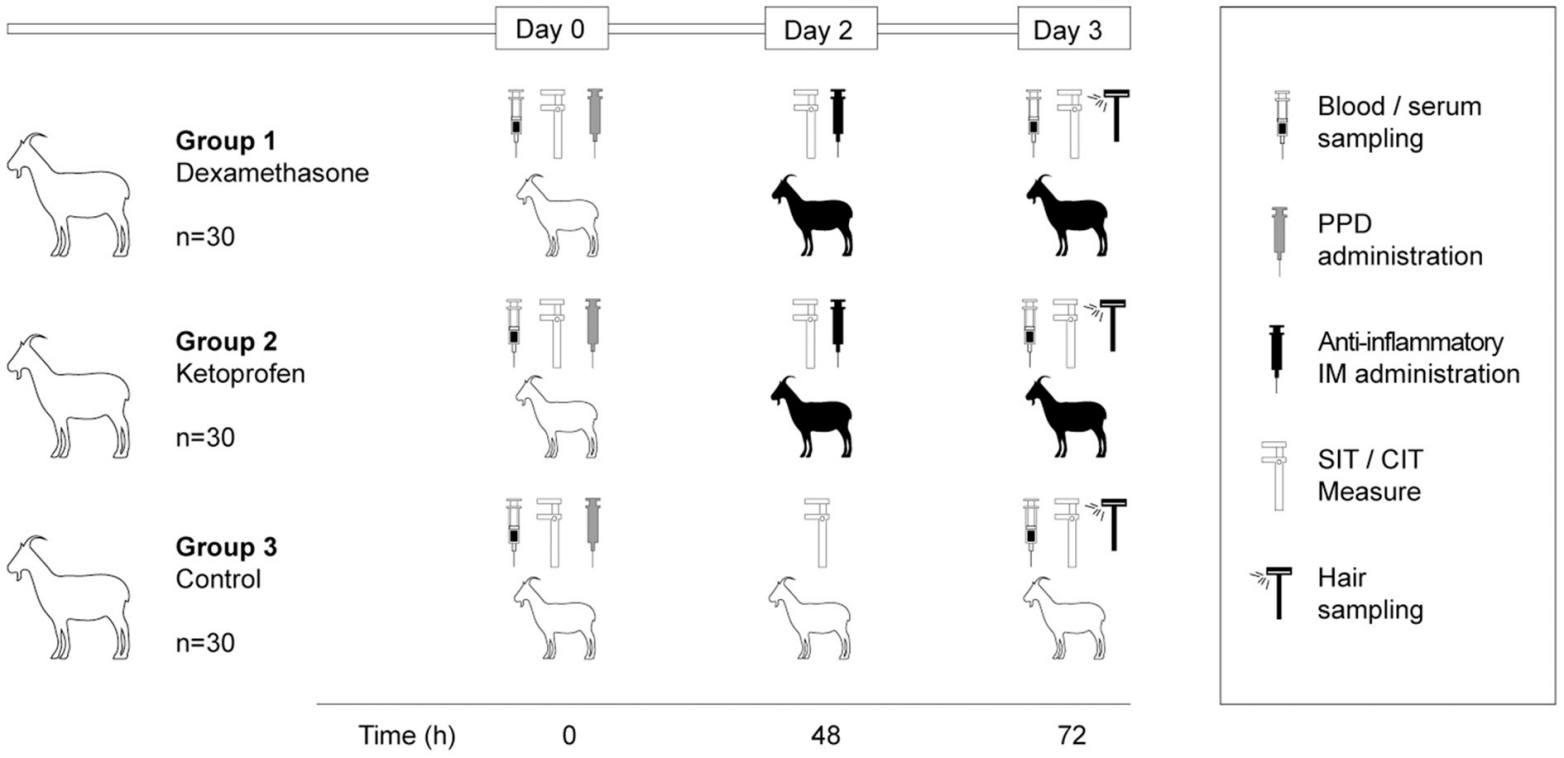
Summary of the experimental design. White silhouettes represent goats untreated and black goat silhouettes represent goats treated with dexamethasone (0.06 mg/kg) or ketoprofen (3 mg/kg).

The study was carried out in a TB-infected dairy goat herd of the Guadarrama breed located in central Spain. This herd (n = 161) had previously been confirmed as TB-infected by means of bacteriological culture (*M. bovis* SB0121). The animals were subjected to SIT test, CIT test, IGRA (in plasma samples) and P22 ELISA (in serum samples) 3 months before the start of this study and there was an apparent prevalence of 40.9% (95% CI 33.7–48.7), 7.5% (95% CI 4.3–12.6), 50.3% (95% CI 42.7–57.9) and 43.5% (95% CI 36.1–51.2), respectively. According to these results, this herd was considered a high prevalence herd. Furthermore, this herd had undergone a vaccination programme against *Mycobacterium avium* subsp. *paratuberculosis* (MAP), using the Gudair vaccine (CZ Vaccines, Porriño, Spain) in 6-months old animals. The goats were selected on the basis of previous TB testing results using SIT test and IGRA in parallel. The animals (*n* = 90) were randomly distributed in three experimental groups: (1) treated with dexamethasone (*n* = 30); (2) treated with ketoprofen (*n* = 30) and (3) control (*n* = 30) groups (Figure 1). All the animals were subjected to the SIT and CIT tests by means of the intradermal injection of bovine and avian PPDs (day 0), and two readings were conducted: one at day 2 (48 h after the PPDs injections) and the official reading at day 3 (72 h after the PPDs injections). Just after the first reading, the recommended doses (in a final volume of 1.5 ml) of dexamethasone (Dexamethasone Sodium Phosphate 2 mg/ml, Cenmetasona, CENASIVA S.L., Reus, Spain) or ketoprofen (Ketoprofen 100 mg/ml, Ketink, Industrial Veterinaria S.A., Barcelona, Spain) were administered intramuscularly on the left side of the neck in the dexamethasone and ketoprofen groups, respectively (Figure 1). IGRA and P22 ELISA were performed using plasma and serum, respectively, both obtained from whole blood samples collected at days 0 and 3 (Figure 1). Moreover, hair and serum samples were collected at day 3 (Figure 1) in order to detect dexamethasone and ketoprofen using High Performance Liquid Chromatography-Mass Spectrometry (HPLC-MS). Razor blades were used to collect fifteen hair samples from the left and right sides of the neck and another 15 from the left and right sides of the backs of the animals from the dexamethasone, ketoprofen and control groups. Serum samples were collected from all the animals in each experimental group. In addition, serum samples from 10 animals in each group were assayed for the analysis of the cytokine/chemokine production pattern in the three experimental groups.

All handling, testing and sampling procedures were carried out by qualified veterinarians in compliance with European (86/609/CEE) and Spanish (RD 53/2013) legislation. The procedures employed in the current study were similarly approved by an institutional ethical committee and ratified by the local authority (PROEX11/18; Comunidad de Madrid).

### Intradermal tuberculin tests

The animals were subjected to SIT and CIT tests, which were carried out by intradermally inoculating them with 0.1 ml of bovine and avian PPDs (CZ Vaccines, Porriño, Spain) using a Dermojet syringe (AkraDermojet, France) on the left-medial and right-medial side of the neck, respectively. The intradermal inoculation of PPDs were carried out at day 0 in all groups and the reactions were interpreted 48 h (day 2) and 72 h (day 3) later by the same veterinarian (Figure 1). SIT and CIT tests were performed according to the protocol published by the European Union Reference Laboratory (EU-RL) for bovine TB following Regulation EU 2016/429, the Commission Delegated Regulation EU 2020/688 and Royal Decree 2611/1996. The interpretation of results was carried out as described previously (20).

### Interferon-gamma release assay

Blood samples were collected from the jugular vein by venepuncture using evacuated tubes (BD Vacutainer Becton, Dickinson and Company, Franklin Lakes, USA) with lithium heparin at days 0 and 3 for the detection of IFN-γ production (Figure 1). In the laboratory, the blood samples were stimulated with bovine and avian PPDs (CZ Vaccines, Porriño, Spain) at a final concentration of 20 μg/ml each and were processed as described elsewhere (11). IFN-γ production in plasma was measured using a commercial kit (Bovigam TB, Thermo Fisher Scientific, Waltham, USA) following the manufacturer's instructions, and the results were interpreted as described previously (21).

### P22 ELISA

Specific antibodies against the MTBC were detected using an in-house indirect enzyme-linked immunosorbent assay based on the multiprotein complex P22 (P22 ELISA) obtained by the inmunopurification of bovine PPD (CZ Vaccines, Porriño, Spain). The bovine and avian PPDs traditionally used for TB diagnosis in ruminants contain 456 and 1019 proteins respectively, reduced to 118 in P22. There are 143 proteins that are present both in bovine and avian PPDs, potentially responsible of the cross reactivity, which are reduced to 43 in the P22 complex, explaining the higher specificity obtained using P22 (22). Blood samples were collected from the jugular vein using plastic serum tubes (BBD Vacutainer Becton, Dickinson and Company, Franklin Lakes, USA) at days 0 and 3 (Figure 1). The ELISA was performed on these serum samples as described previously (23). The serum sample results were expressed as an ELISA percentage (E%), calculated according to the following formula:

Sample E% = [mean sample OD / (2 x mean of negative control OD)] × 100

Serum samples with E% values >150 or 100 were considered positive to the standard and severe interpretations, respectively, as described elsewhere (20, 23).

### High performance liquid chromatography-mass spectrometry

Dexamethasone (purity > 98%), ketoprofen (purity > 99%), formic acid (purity > 99% for analysis) and sodium acetate (purity > 98% for analysis) were purchased from Sigma-Aldrich (St. Louis, MO, USA), while the different organic solvents (HPLC grade) were obtained from Merk (Darmstadt, Germany). The water employed in the mobile phase and for the sodium solution 1M was obtained from Milli-Q water from Millipore system (Bedford, MA, USA). Nitrogen generated by a nitrogen generator obtained from Peak Scientific Instruments, Ltd. (Chicago, IL, USA) was employed for MS detection and solvent evaporation. Stock solutions of dexamethasone (0.8 mg/mL) and ketoprofen (0.8 mg/ml) were prepared in methanol (Merk, Darmstadt, Germany) and stored in the dark at −18°C. These stock solutions of each substance were mixed several times with methanol to obtain a working standard solution mixture of the selected drugs at 1 and 0.1 μg/ml.

The serum and hair samples of the dexamethasone and ketoprofen groups were analyzed in an LC-MS/MS system (Bruker Bremen, Germany). Analytes were separated on an Acquity UPLC BEH C18 chromatographic column (2.1 × 100 mm, 1.7 μm) obtained from Waters (Milford, USA) for dexamethasone detection and on an Intensity Solo HPLC column C18 (2.0 × 100 mm, 2 μm) for ketoprofen, also from Bruker Daltonik. The separation of the analytes was achieved for both substances in a gradient mode phase A mixture (0.1% acidified water with formic acid) and a phase B mixture (methanol 0.1% acidified with formic acid for ketoprofen and acetonitrile 0.1% acidified with formic acid for dexamethasone).

#### HPLC-MS in serum samples

Five hundred μl of serum sample was transferred into 2 ml disposable Eppendorf tubes, and 1.5 ml of acetonitrile (ketoprofen) and 800 μl of acetonitrile (dexamethasone) (Merk, Darmstadt, Germany) were added. Analyte-free goat serum samples were spiked with ketoprofen to 20, 50, 100, 150 and 300 ng/ml or with dexamethasone to 1, 5, 10, 25, 50 and 100 ng/ml for quantification. The limit of detection was 10 ng/sample for the detection of dexamethasone and ketoprofen in serum samples.

#### HPLC-MS in hair samples

The ketoprofen and dexamethasone were extracted as described previously for betamethasone (16). Fifteen μl of the extract was then injected into the HPLC column for analysis by means of HPLC-MS/MS. Each day of analysis, calibration curves were prepared with 5 ml of acetonitrile in 15 ml Falcon tubes, to which different amounts of ketoprofen were added in order to obtain a final concentration of 0.1, 0.2, 2, 5, and 10 ng/ml. In the case of dexamethasone, in order to quantify the pharmaceutical, each day of analysis, buffer solutions were spiked with dexamethasone to final concentrations of 0.1, 0.5, 1, 20, 40, and 80 ng/ml, the extraction protocol was applied, and the peak area recorded. Equally to serum samples, the limit of detection was 10 ng/sample of both substances in hair samples.

### Cytokine/chemokine production pattern detection

A Luminex-based cytokine/chemokine array detecting 15 cytokines and chemokines was applied to the serum samples collected at day 3 from 10 goats randomly selected from each group. The cytokines/chemokines present in the serum samples were then quantified using a bovine multiplex assay kit (MILLIPLEX MAP Kit Bovine Cytokine/Chemokine Magnetic Bead Panel 1, 96-Well Plate Assay, Merck Millipore, UK). Serum samples were centrifuged at 10,000 g for 5 min. The serum was subsequently diluted (1:2 in the assay buffer provided in the kit). Twenty-five μl of each mix (serum and buffer) were then analyzed in duplicate on Bio-Plex^®^ 200 (Bio-Rad Laboratories, S.A., Alcobendas, Spain) in order to determine the amount (expressed in pg/ml) of IFN-γ, IL-1α, IL-1β, IL-6, IL-8, IL-17A, IL-36RA, IP-10, MCP-1, MIP-1α MIP-1β, TNFα VEGF-A (proinflammatory response), IL-4 and IL-10 (anti-inflammatory response) in the serum samples. This result was the mean of two analyses of each sample and multiplied by the dilution factor.

### Statistical analysis

Data analyses were performed using SPSS Statistics 25 (IBM, New York, NY, USA), and R version 4.0.5 software (24) and interpreted by using a *p*-value of 0.05 to determine statistical significance. Wilson's 95% confidence intervals (95% CI) were calculated for the percentage of reactors to the different diagnostic techniques using WinPepi version 11.6 (25). The comparison of the proportions of test reactors within a given group between days 0 and 3 (IGRA and P22 ELISA) and 2 and 3 (intradermal tuberculin tests) was performed by using McNemar's test. The comparison of the proportions of test reactors among the different groups at a given sampling point was performed using a logistic regression model with the test result (positive/negative) as the outcome variable and the experimental group (dexamethasone/ketoprofen/control as reference category) as the predictor. Quantitative differences in the increase in skin fold thickness (SFT), optical density (OD) and E% between days 0, 2 and 3 were analyzed by means of the Wilcoxon signed-rank test. Quantitative values, such as the increase in the SFT (expressed in mm), IFN-γ levels (OD), ELISA percentage (E%) and the amount of cytokine/chemokines (expressed in pg/ml) in the goats in the different groups at a given sampling point were compared using the Kruskal-Wallis test followed by pairwise tests for multiple comparisons of mean rank sums after adjusting the *p*-value using the Bonferroni correction. With regard to IFN-γ levels, Spearman's rank correlation coefficient (r_s_) was used to assess the relationship between ODs in plasma and the pg/ml of cytokine observed in serum samples. Heatmap clustering analysis of cytokine/chemokines in serum samples was conducted using an online tool (Heatmapper, 164 www.heatmapper.ca) (26).

## Results

### Cell-based diagnostic tests

**Figure 2:**
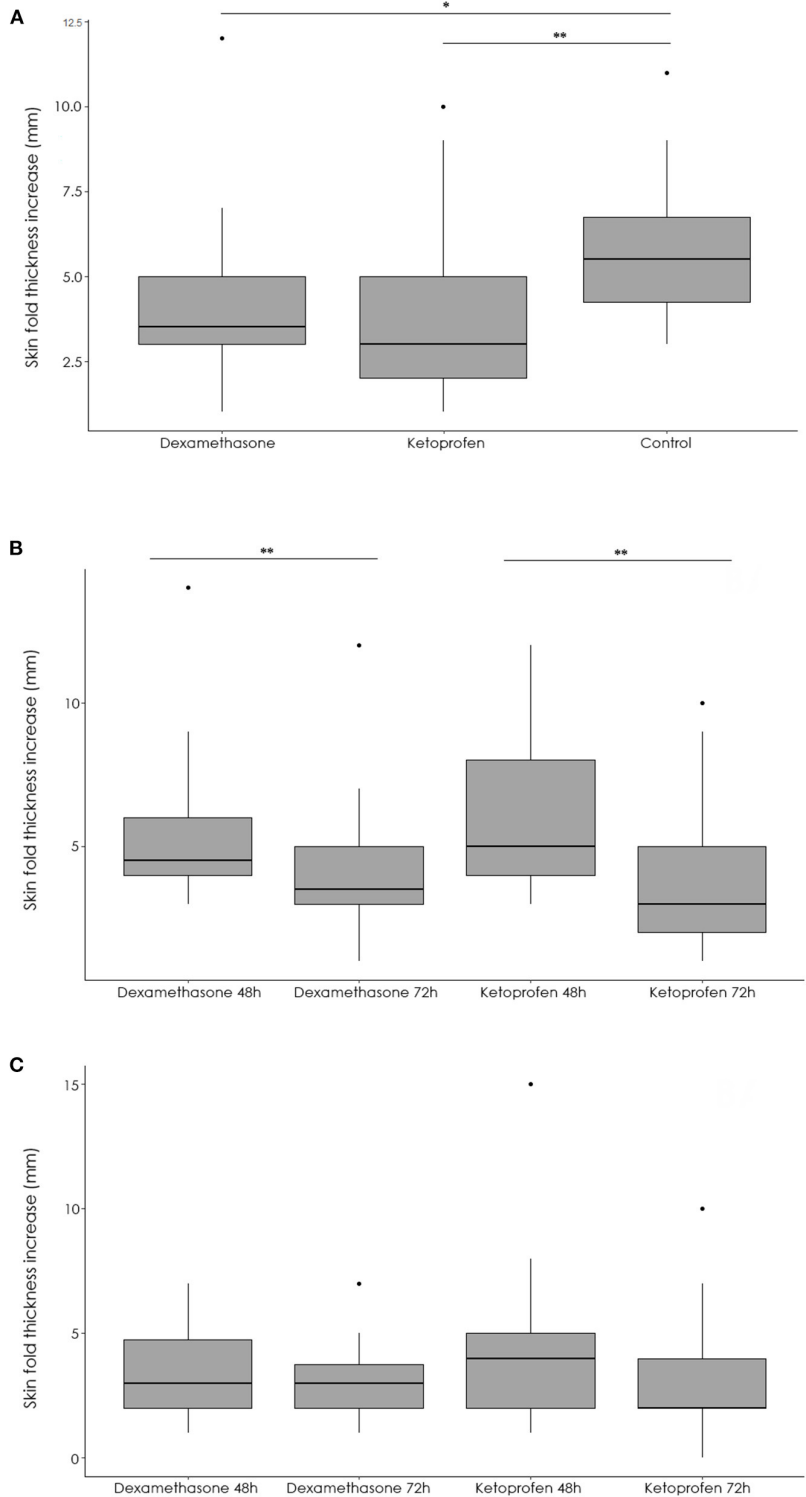
Summary of the differences among median skin fold thickness (mm) after bovine PPD injection in the dexamethasone, ketoprofen and control groups at day 3 **(A)**, and after bovine **(B)** and avian **(C)** PPD injection in the dexamethasone and ketoprofen groups at 48 h and 72 h. *p = 0.005, **p < 0.001.

The number and percentage of positive reactors obtained using the different diagnostic tests are summarized in Table 1. While in day 2 (before the inoculation of the drugs) there was no significant (*p* = 0.27) association between the group and the test result, the probability of finding a SIT test reactor was significantly lower (*p* < 0.001) in the dexamethasone (OR = 0.11, 95% CI 0.02–0.40) and ketoprofen (OR = 0.08, 95% CI 0.01–0.30) groups than in the control group at day 3 after the bovine PPD inoculation. This finding was due to a lower increase in the SFT in the dexamethasone (*p* = 0.005) and ketoprofen (*p* < 0.001) groups when compared to the control group (Figure 2A).

**Table 1:** Number of positive reactors (including percentage and Wilson's CI 95%) to diagnostic tests in each experimental group.

		**Day 0 (** ***N*** **, %** ^**e**^ **)**				**Day 2 (** ***N*** **, %** ^**e**^ **)**				**Day 3 (** ***N*** **, %** ^**e**^ **)**							
**Group**	**n**	**IGRA 0.05^a^**	**IGRA 0.1^a^**	**P22 100^b^**	**P22 150^b^**	**SIT test** ^**c**^		**CIT test** ^**d**^		**SIT test** ^**c**^		**CIT test** ^**d**^		**IGRA 0.05^a^**	**IGRA 0.1^a^**	**P22 100^b^**	**P22 150^b^**
						**Standard**	**Severe**	**Standard**	**Severe**	**Standard**	**Severe**	**Standard**	**Severe**				
Dexamethasone	30	5, 16.7 (7.3–33.5)	3, 10.0 (3.5–25.6)	13, 43.3 (27.4–60.8)	11, 36.7 (21.8–54.5)	28, 93.3 (78.7–98.2)	30, 100 (88.6–100)	7, 23.3 (11.8–40.9)	21, 70.0 (52.1–83.3)	15, 50.0 (33.2–66.8)	24, 80.0 (62.7–90.5)	5, 16.7 (7.3–33.5)	13, 43.3 (27.4–60.8)	12, 40.0 (24.6–57.7)	8, 26.7 (14.2–44.5)	16, 53.3 (36.1–69.8)	14, 46.7 (30.2–63.9)
Ketoprofen	30	7, 23.3 (11.8–40.9)	3, 10.0 (3.5–25.6)	14, 46.7 (30.2–63.9)	12, 40.0 (24.6–57.7)	27, 90.0 (74.4–96.5)	30, 100 (88.6–100)	11, 36.7(21.8–54.5)	21, 70.0 (52.1–83.3)	13, 43.3 (27.4–60.8)	17, 56.7 (39.2–72.6)	6, 20.0 (9.5–37.3)	14, 46.7 (30.2–63.9)	8, 26.7 (14.2–44.5)	4, 13.3 (5.3–29.7)	15, 50.0 (33.2–66.8)	13, 43.3 (27.4–60.8)
Control	30	10, 33.3 (19.2–51.2)	6, 20.0 (9.5–37.3)	16, 53.3 (36.1–69.8)	15, 50.0 (33.2–66.8)	24, 80.0 (62.7–90.5)	27, 90.0 (74.4–96.5)	5, 16.7 (7.3–33.5)	20, 66.7(48.8–80.7)	27, 90.0 (74.4–96.5)	30, 100 (88.6–100)	2, 6.6 (1.8–21.3)	15, 50.0 (33.2–66.8)	13, 43.3 (27.4–60.8)	9, 30.0 (16.7–47.9)	19, 63.3 (45.5–78.1)	16, 53.3 (36.1–69.8)

^a^An animal was considered positive to the IGRA if the optical density (OD) of a sample stimulated with bovine PPD minus the OD of PBS was >0.1 (standard interpretation) or 0.05 (severe interpretation) and greater than the OD of the sample stimulated with avian PPD.

^b^An animal was considered positive to P22 ELISA when the E% value was >150 (standard interpretation) or 100 (severe interpretation).

^c^An animal was considered a positive reactor to the SIT test when there was an increase of ≥ 4 mm (standard interpretation) or >2 mm (severe interpretation) in the skin fold thickness and/or the presence of clinical signs was observed.

^d^An animal was considered a positive reactor to the CIT test when the bovine reaction was greater than the avian reaction by more than 4 mm (standard interpretation) or the bovine reaction was ≥3 mm and greater than the avian reaction (severe interpretation); and/or there were clinical signs at the bovine PPD inoculation site.

^e^Wilson's 95% confidence intervals.

**Figure 3:**
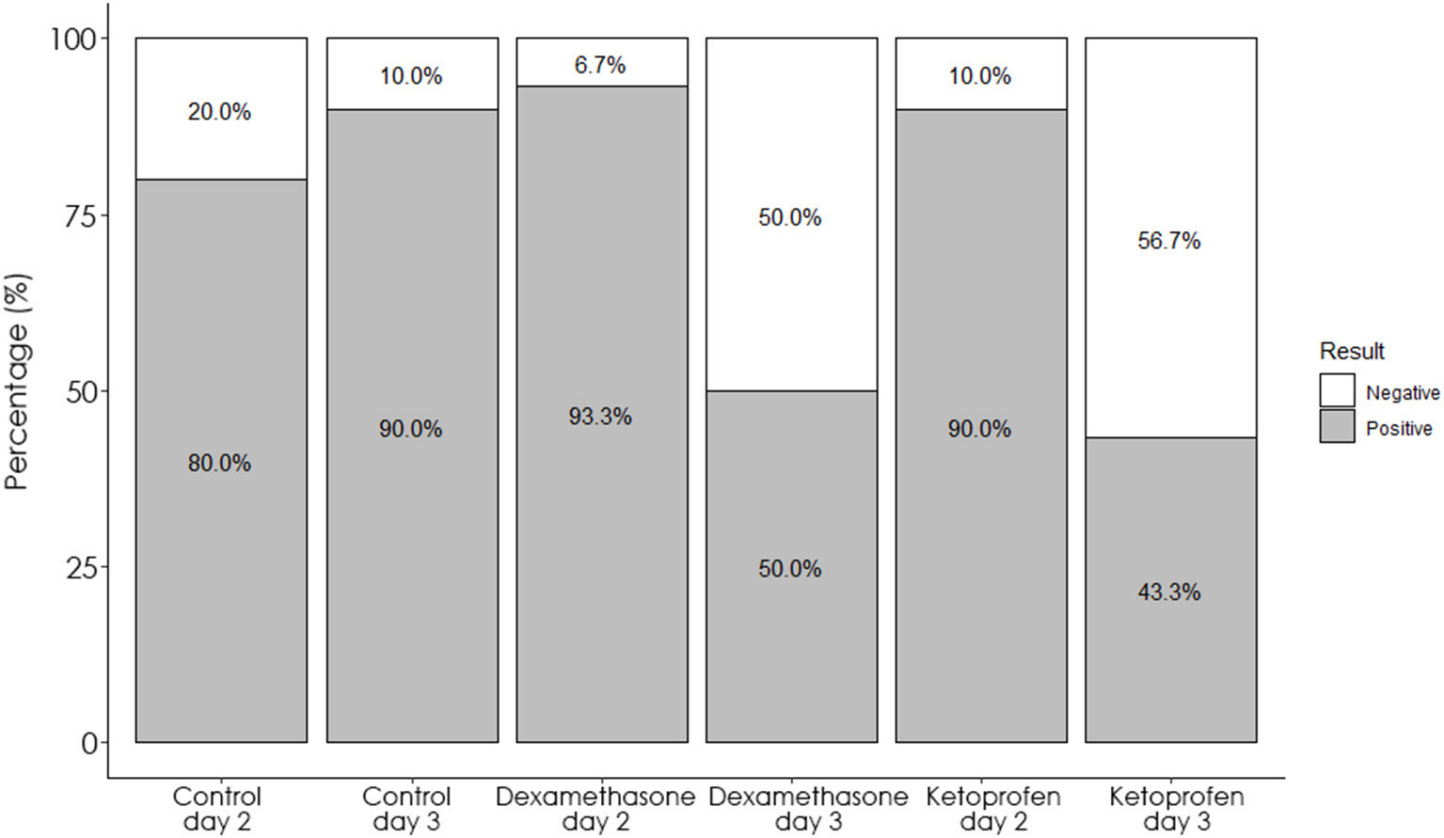
Percentage of animals that were positive (gray) and negative (white) to SIT test in the dexamethasone, ketoprofen and control groups at day 2 and day 3.

When measures obtained in day 2 (48 h, time of application) and 3 (72 h, time of SIT test interpretation) for each experimental group were compared, the SFT at the bovine inoculation site was reduced in both dexamethasone and ketoprofen groups (Figure 2B). This reduction in the SFT led to a significant (*p* < 0.001) decrease in both the number of reactors at day 3 respected to those observed at day 2 using standard interpretation of the SIT test (Table 1, Figure 3). Interestingly, the reductions in the SFT at the avian PPD inoculation site were not significant in the dexamethasone (*p* = 0.085) and ketoprofen (*p* = 0.05) groups between day 2 and day 3 (Figure 2C).

In contrast, a significant increase in the SFT at the bovine (*p* = 0.002) and avian (*p* = 0.01) PPD inoculation site between day 2 and day 3 was observed in the control group. Moreover, the increase of the SFT was significantly higher at the bovine PPD inoculation site compared to the avian PPD inoculation site in the dexamethasone (*p* = 0.01) and ketoprofen (*p* = 0.006) groups. In this line, the reduction in the number of reactors to the CIT test was not significant in the dexamethasone (*p* = 0.50), and ketoprofen (*p* = 0.06) groups between day 2 and day 3 when using the standard interpretation of the CIT test (Table 1). The increase in the SFT was significantly (*p* < 0.001) higher at bovine PPD inoculation site compared to those reactions observed at the avian PPD inoculation site in the reactors to CIT test when using standard interpretation 48 h after the PPDs administration in the three groups.

**Figure 4:**
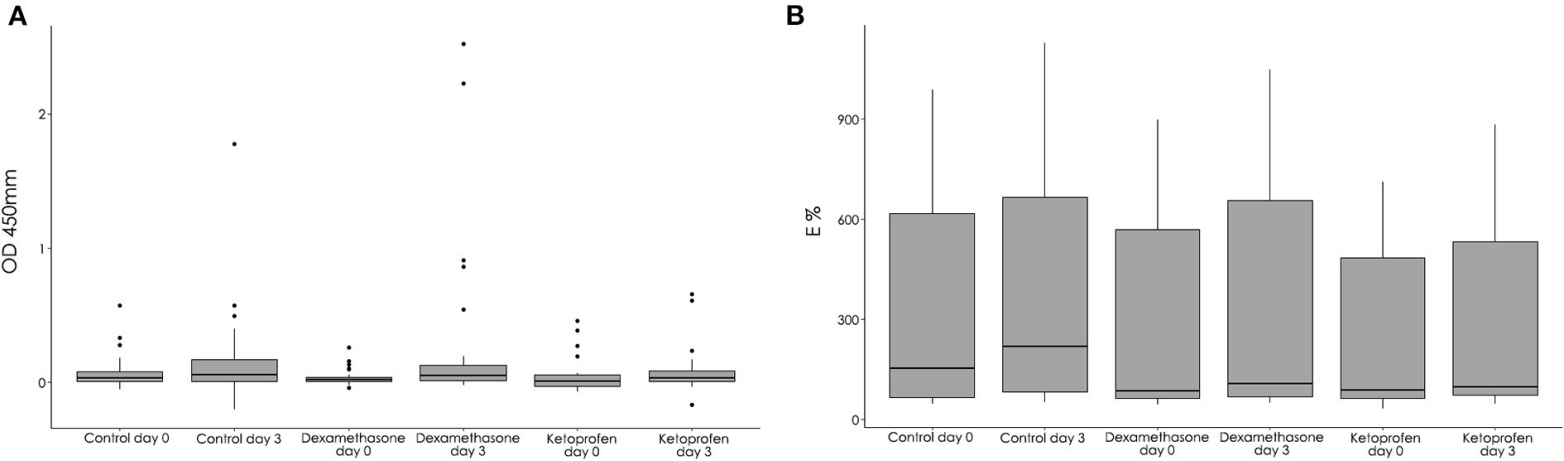
Interferon-gamma release assay (IGRA) values expressed as optical density (OD) in the dexamethasone, ketoprofen and control groups at day 0 and day 3 **(A)** and P22 ELISA values expressed as ELISA percentage (E%) in the dexamethasone, ketoprofen and control groups at day 0 and day 3 **(B)**.

The intramuscular injection of dexamethasone and ketoprofen was not associated with the IGRA results, since no significant (*p* = 0.35) association between the group and the test result was observed (dexamethasone: OR = 0.87, 95% CI 0.31–2.44; ketoprofen: OR = 0.47, 95% CI 0.15–1.38) in day 3 using the control group as a reference. Similarly, the differences between the IFN-γ results (OD) at day 3 in the groups were not significant (*p* = 0.47, Figure 4A). A significantly higher number of IGRA positive reactors (*p* = 0.01) was detected within the dexamethasone group when the test was performed at day 3 when compared to day 0 (Table 1). With regard to the quantitative results, a significant increase (*p* = 0.02) in the IFN-γ measures was observed between day 0 and day 3 in the dexamethasone group. However, no significant differences in the IGRA results (ODs) were observed between day 0 and day 3 in the control (*p* = 0.58) and ketoprofen (*p* = 0.06) groups.

### Antibody-based diagnostic test

The recent intramuscular administration of dexamethasone or ketoprofen was not associated with the result in the P22 ELISA, since the differences between the groups in terms of quantitative (E%) and qualitative (positive reactors) were not significant at day 3 (*p* = 0.52 and *p* = 0.55, respectively). In this respect, a significant increase in the E% was observed between day 0 and day 3 in the dexamethasone (*p* < 0.001), ketoprofen (*p* = 0.001) and control (*p* < 0.001) groups (Figure 4B). However, this significant increase in the E% did not lead to a significant increase in the number of positive goats between day 0 and day 3 using standard (*p* = 0.25, *p* = 1 and *p* = 1 in dexamethasone, ketoprofen and control groups, respectively) or severe interpretation (*p* = 0.25, *p* = 1 and *p* = 0.25 in dexamethasone, ketoprofen and control groups, respectively).

### Cytokine/chemokine production pattern detection

**Figure 5:**
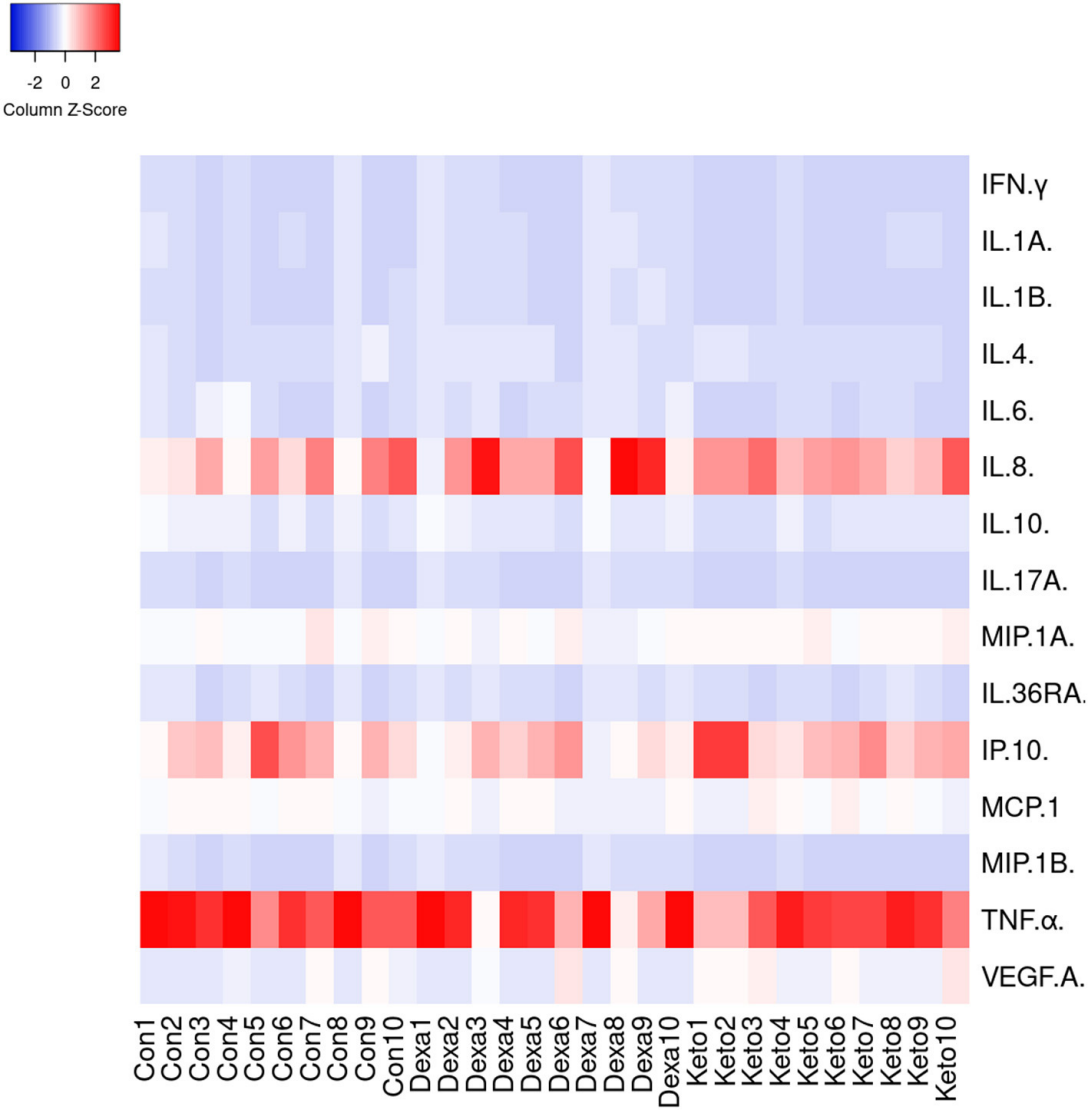
Heatmap of clustering analysis for different cytokines and chemokines analyzed in serum samples collected at day 3 from 10 goats randomly selected from each group (26).

A similar pattern was observed for the 15 cytokines and chemokines measured on day 3 (Figure 5). No significant differences (*p* > 0.05) among the different groups were observed in terms of the amount (pg/ml) of each cytokine/chemokine in the serum samples. Twelve out of the fifteen cytokines analyzed were detected in all animals, regardless of the group. However, IL-1β was detected in 9 out of 30 goats, whereas IL-17A and MIP-1β were detected in 16 animals. Nevertheless, this lack of detection was not associated with a specific group. In contrast, high levels of IL-8, IP-10 and TNFα were observed in serum samples taken from animals from all the groups (Figure 5), thus suggesting a stronger basal proinflammatory response to the TB infection in these animals, regardless of the group. Finally, the correlation (Spearman's rank correlation coefficient) between the amount of IFN-γ in plasma samples observed after the bovine PPD stimulation (OD) and the levels of this cytokine in serum samples analyzed using the multiplex assay was poor (r_s_ = 0.215).

### Dexamethasone and ketoprofen detection by HPLC

The presence of residues of dexamethasone and ketoprofen in hair and serum samples collected at day 3 was not detected when employing the HPLC-MS. A minimal amount of residues of this substances were observed in the hair samples of only four animals in the dexamethasone group. Nevertheless, the amount detected were far below of the cut-off usually applied for official analysis (10 ng/ml), ranging from 0.85 to 1.4 ng/sample.

## Discussion

The parenteral administration of dexamethasone or ketoprofen 48 h after the PPDs inoculation in these experimental groups significantly reduced the increase in SFT when compared to the control group. This reduction, therefore, led to an increase in false negatives results to the intradermal tests. In this respect, several previous studies have evidenced the effect of different anti-inflammatory substances on the immunological response in cattle (17, 18, 27–29). Some of these studies have evaluated the effect of dexamethasone on the TB diagnosis techniques in cattle, such as the intradermal tuberculin test (17) and IGRA (17, 18). Other previous studies have evaluated the *in vitro* effect of dexamethasone, meloxicam or flunixin meglumine on different cell populations in cattle (27–29). However, to the best of the authors' knowledge, this is the first study to report the effect of the administration of a recent parenteral corticosteroids and non-steroid anti-inflammatory substances on different antemortem TB diagnostic techniques used in goats. In this study we have demonstrated the capacity of dexamethasone and ketoprofen recently administered to alter the caprine TB diagnosis by considerably reducing the number of reactors to SIT test. Our results are supported by a previous study of Doherty and collaborators in which a significant reduction in the SFT was observed in TB-infected cattle treated with dexamethasone when compared with untreated control cattle (17). The main difference between the aforementioned study with cattle and our experiment was the administration strategy employed. The parenteral administration of dexamethasone or ketoprofen 48 h after the PPDs administration demonstrated the potential use of these substances with fraudulent purposes. In this sense, the authorities of different regions in Spain have started to research the possible parenteral administration with fraudulent purposes using different substances such as anti-inflamatory substances in the recent years (19). There is a high variety of substances and administration protocols which can be applied in ruminants in order to modify the results of the TB diagnostic tests and a lack of knowledge of its effect on the *in vitro* techniques. Up to date, there is no evidence to recommend IGRA or diagnostic tests based on the humoral response to avoid the interferences caused by fraudulent activities on the intradermal tests. Recently, IGRA was included as official test for diagnosis of caprine animals in the context of the New Animal Health Law (Regulation 2016/429) and therefore, it would be valuable to evaluate the effect of different fraudulent strategies on IGRA results in goats.

The fraudulent activity evaluated in the present study could, therefore, be directed toward specific animals (those showing evident reactions at 24–48 h) rather than of all the animals tested. It is necessary to highlight that in our study, the increase in the SFT was even lower at the bovine PPD inoculation site when compared to the avian PPD inoculation site, probably as a result of the administration of ketoprofen and dexamethasone at the same side of the neck as the bovine PPD. According to these findings, the dexamethasone or ketoprofen inoculation site affected the SIT test results, since the significant decrease in the SFT was observed only at bovine PPD inoculation site (left-medial side of the neck in our study). This could, therefore, explain the significant reduction in reactors to the SIT test in the present study. However, the parenteral administration of dexamethasone or ketoprofen did not lead to a significant reduction in the number of reactors to CIT test, probably owing to the significant higher SFT at the bovine PPD inoculation site compared to those observed at the avian PPD inoculation site in the dexamethasone and ketoprofen groups the day of the intramuscular administration (48 h after the PPDs injection). This fact and the reduction in the SFT observed at the avian PPD inoculation site in certain animals could explain the lack of a significant reduction in the reactors to CIT test in all groups.

With regard to the effect that the intramuscular inoculation of anti-inflammatory substances had on the IGRA results obtained for animals treated with anti-inflammatory drugs, previous studies have shown that the intramuscular administration of dexamethasone reduced the IFN-γ levels observed in TB-infected cattle (17, 18). Nevertheless, under the conditions of our study, the intramuscular inoculation of dexamethasone or ketoprofen did not lead to a significant effect on the number of animals that tested positive to IGRA since the differences in the number of reactors observed in the dexamethasone or ketoprofen groups and the control group were not significant, probably due to the administration strategy and dose used. The increase in the quantitative values (OD), and subsequently, the number of reactors to IGRA that were observed are probably owing to a booster effect associated with the recent previous PPD administration in the dexamethasone and ketoprofen groups. These results are in agreement with a previous study in TB-infected goats that demonstrated that a recent previous PPD inoculation had a significant effect on IGRA results by increasing the number of reactors when blood was collected 3 days after the intradermal test (16). Nevertheless, this effect was not observed in the control group, and is probably associated with the high number of reactors to IGRA at day 0 in the control group when compared to dexamethasone group. Another possible reason that could explain this finding might be the posology used: in the study with cattle, blood was collected from both groups 72 and 36 h after dexamethasone (0.2 mg/kg) administration, respectively, whereas in our study, dexamethasone (0.06 mg/kg) was applied 24 h before blood collection (17). The dose used in our study was considerably lower than that used in cattle, suggesting the possibility that higher doses could have a similar effect on goats to those observed in cattle (17, 18). With regard to the absence of effect of ketoprofen on IGRA outcome, a previous *in vitro* study showed demonstrated that non-steroid anti-inflammatory substances such as meloxicam did not reduce the IFN-γ produced by lymphocytes in cattle, unlike dexamethasone (29). In this respect, it is necessary to highlight that the *in vitro* study, which showed an evaluation of the effects of dexamethasone and meloxicam on lymphocyte, employed groups of samples incubated in the absence (controls) or presence of dexamethasone or meloxicam, and this evaluation was performed *in vivo* in our study (29). Our results consequently showed the lack of effect of ketoprofen on the IGRA results in goats, at least under the conditions of our study.

The differences between the number of positives to P22 ELISA in the dexamethasone group and in the control group and between the ketoprofen group and the control group were not significant in the present study regardless the cut off employed (100 or 150 %E), probably owing to the single administration performed 24 h before these animals were sampled. A recent parenteral inoculation of dexamethasone or ketoprofen did not, therefore, have an immunosuppressive effect on the humoral response. In fact, a significant increase in the Ab levels, expressed as E%, when using P22 ELISA was detected in the three study groups 3 days after the PPD was administered. This booster effect has been described previously and is used in domestic ruminants to increase the sensitivity of the skin tests in specific circumstances (30–35). Although the Ab titres and, therefore, the sensitivity of the serial use of serological tests are maximized between 15 or 30 days after the PPD intradermal inoculation, the increase in the Ab titres 3 days after the skin test that we describe in our study has been observed previously in goats (36).

The recent parenteral administration of the dexamethasone and ketoprofen did not significantly affect the cytokine/chemokine profile evaluated, since the differences in the levels of any cytokine/chemokine observed in serum samples obtained from the dexamethasone and ketoprofen groups were not significant with respect to those observed in animals from the control group. Our study showed two cytokines and one chemokine, which were infrequently detected and at low concentrations in the serum samples, regardless of the group: IL-1β, MIP-1β and IL-17A. These results are similar to those observed in a previous study in cattle, in which low concentrations of IL-1β and IL-17A were observed in interstitial fluid and plasma samples (37). However, these results were unexpected, since both cytokines have previously been described as biomarkers of *M. bovis* infection (38, 39). With regard to MIP-1β, the low concentration observed is contrary to that of a previous study in cattle in which high plasma levels were observed (37) and could be associated with differences between cattle and goats. In our study, IL-8, IP-10 and TNFα were detected at high concentrations in serum samples. In this respect, previous studies have shown the potential use of IL-8 (40), IP-10 (41–43) and TNFα (39, 44) as biomarkers of TB-infection in cattle. It is necessary to highlight that the low levels of IL-1β, MIP-1β and IL-17A and the high levels of IL-8, IP-10 and TNFα were not associated with a specific group and were observed in all apparent *M. bovis* infected goats (reactors). The higher concentrations of IL-8, IP-10 and TNFα (proinflammatory response) when compared to IL-4 or IL-10 (anti-inflammatory response) suggested a induction of proinflammatory response due to the infection in these TB-infected animals, regardless of the study group. With regard to IFN-γ, the poor correlation observed between the IGRA results (OD) and the levels of this cytokine in serum samples was probably associated with the previous PPD stimulation of whole blood samples during the performance of IGRA.

Previous studies have demonstrated that a high variety of substances and administration protocols can be applied in ruminants in order to modify the results of the TB diagnostic tests (16–18). This complicates the detection of these fraudulent activities. The development and standardization of tools to detect these substances is, therefore, essential in order to prevent those activities that affect advances in TB eradication programmes in ruminants. In this respect, it is important to highlight that the administration method affects the detection of substances used with fraudulent purposes, as shown in a previous study (16). Our study demonstrates that certain anti-inflammatory drugs, such as dexamethasone and ketoprofen, cannot be detected on hair and serum samples when using the HPLC technique if they are intramuscularly administered. However, the detection of betamethasone topically applied at the site of PPD injection using the HPLC technique on hair samples collected from the PPD inoculation site have attained excellent result previously (16). Using these previous results as a basis, we decided to use hair samples in this study, since this sample is easy to collect by using an ante-mortem and non-invasive method. Moreover, the time of application was the same in both studies: 48 h after PPD inoculation (16). The detection of anti-inflammatory substances, therefore, depends on the administration method (topical *vs*. parenteral), and it is useful in the case of topical administration. However, the significant reduction in the increase in the SFT observed in the dexamethasone and ketoprofen groups led to a reduction in the number of reactors to the intradermal test and, therefore, to an increase in false negatives to the intradermal tests. In this context, it is necessary to highlight that the lack of detection of these substances does not imply the absence of its effect on the caprine TB diagnostic technique results, since the reduction in the increase in SFT was observed in animals in whose hair or serum samples the substances were not detected.

In conclusion, the present study has, for the first time, evaluated the effect of the recent intramuscular administration of dexamethasone and ketoprofen on the results of the SIT and CIT tests, IGRA, and P22 ELISA obtained in goats. Our study demonstrates that corticosteroids and non-steroid anti-inflammatory substances can modify the SIT test results in goats when they are inoculated 48 h after the PPD administration. Nevertheless, this recent parenteral administration of the anti-inflammatory substances did not significantly affect the results of the *in vitro* caprine TB diagnostic techniques. The detection of these substances by using HPLC on serum or hair samples was challenging, which could be an issue if they were used fraudulently, since the performance of these fraudulent activities are difficult to demonstrate. Additional studies are, therefore, required in order to develop and optimize an effective protocol to detect these anti-inflammatory substances in different samples.

## Data availability statement

The original contributions presented in the study are included in the article, further inquiries can be directed to the corresponding author.

## Ethics statement

The animal study was reviewed and approved by all handling, testing and sampling procedures were carried out by qualified veterinarians in compliance with European (86/609/CEE) and Spanish (RD 53/2013) legislation. The procedures employed in the current study were similarly approved by an institutional Ethical Committee and ratified by the local authority (PROEX11/18; Comunidad de Madrid). Written informed consent was obtained from the owners for the participation of their animals in this study.

## Author contributions

JO and JB wrote the manuscript, performed the literature search, and designed the figures. JO, CV, and CN performed the experiments. JO, LdJ, IS, JG, ÁR, CV, BR, MD, BP, CN, JS-L, JÁ, and JB interpreted the data. All authors reviewed and approved the manuscript.

## Funding

This study was funded by the Analysis of the long-term caprine tuberculosis eradication process and development of diagnostic tests and control measures for its improvement (GoaTBfree-UCM) Project (PID2019-105155RB-C31). JO was supported by an FPU contract-fellowship (Formación de Profesorado Universitario) from the Ministerio de Ciencia, Innovación y Universidades (FPU18/05197).

## Conflict of interest

The authors declare that the research was conducted in the absence of any commercial or financial relationships that could be construed as a potential conflict of interest.

## Publisher's note

All claims expressed in this article are solely those of the authors and do not necessarily represent those of their affiliated organizations, or those of the publisher, the editors and the reviewers. Any product that may be evaluated in this article, or claim that may be made by its manufacturer, is not guaranteed or endorsed by the publisher.
